# Supplementary material for: Histone variant H2A.Z cooperates with EBNA1 to maintain Epstein-Barr virus latent epigenome
Source: mBio. 2025 Jul 14;16(8):e00302-25. doi: 10.1128/mbio.00302-25 (PMC12345143; doi:10.1128/mbio.00302-25)

SUPPLEMENTARY MATERIALS (For Reviewers Only)

Histone Variant H2A.Z Cooperates with EBNA1 to Maintain Epstein-Barr Virus Latent Epigenome

Leonardo Josué Castro-Muñoz[1], Davide Maestri [1], Leena Yoon [1], Bhanu Chandra Karisetty[1], Italo Tempera[1] and Paul Lieberman[1]

[1]The Wistar Institute, Philadelphia, PA 19104, USA.

*Corresponding Author

lieberman@wistar.org

215-898-9491

Supplementary Figure S1. Lack of Co-Immunopreciptiation of EBNA1 and H2A.Z. **(A)** Mutu I total cell extracts were subject to immunoprecipitation wiutih either antibody to EBNA1 or H2A.Z or control IgG in RIPA buffer extract and wash conditions, followed by Western blot for EBNA1 or H2A.Z. (B) Similar IP as in panel A, but with SNU719.

Supplementary Figure S1

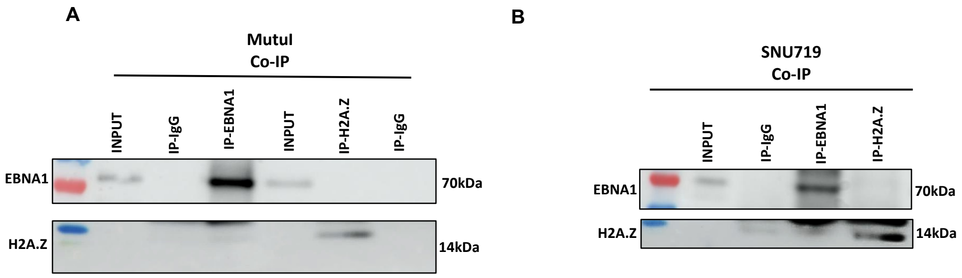

Supplementary Figure S2. Time course of H2A.Z siRNA knockdown on EBNA1 expression and oriP plasmid copy number. A-C) Western blot of FLAG-EBNA1, H2A.Z, β-Actin in 293T cells transfected with CMV-FLAG vector or CMV-FLAG-EBNA1 and co-transfected with siH2A.Z and assayed at either 24 (A), 48 (B), or 72 (C) hrs post-transfection. (**D**) Plasmid copy number determined by qPCR in control (no-CMVFLAG-EBNA1) or after 24, 48, 72 hrs post-transfection with pCMVFLAG-EBNA1-oriP + siH2A.Z).

Supplementary Figure S2

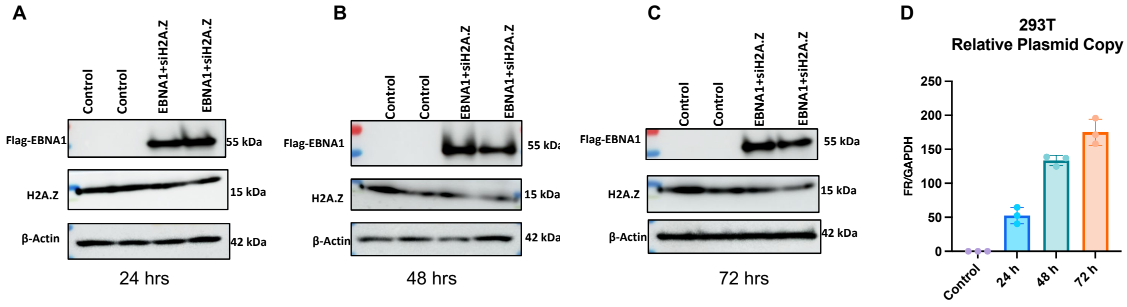

Supplementary Figure S3. Time course of lytic induction in different cells and triggering reagents. (A) Mutu I cells were treated with 20 ng TPA+ 1 mM sodium butyrate (NaB) in Mutu I cells or **(B)** 7.5 μM in SNU719 cells and assayed by Western blot for ZTA, β-Actin, and H2A.Z at 24, 48, or 72 hrs post treatment or with no treatment (control).

Supplementary Figure S3

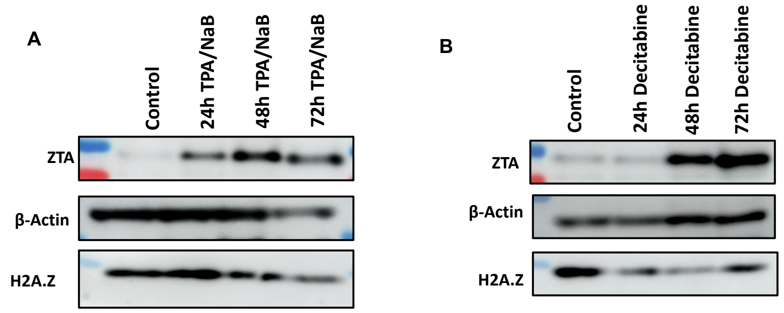

Supplement: Supplemental Figures — Figures S1-S3. [file mbio.00302-25-s0001.pdf]
