# Supplementary material for: Histone variant H2A.Z cooperates with EBNA1 to maintain Epstein-Barr virus latent epigenome
Source: mBio. 2025 Jul 14;16(8):e00302-25. doi: 10.1128/mbio.00302-25 (PMC12345143; doi:10.1128/mbio.00302-25)
Supplement: Table S1 — List of oligonucleotides. [file mbio.00302-25-s0002.pdf]

Table S1. List of qPCR primers and targeting siRNAs

RT-qPCR		
**Name**	**Forward 5´**	**Reverse 3´**
EBNA1	GGTCGTGGACGTGGAGAAAA	GGTGGAGACCCGGATGATG
ZTA	TCTGAACTAGAAATAAAGCGATACAAGAA	TTGGGCACATCTGCTTCAAC
EAD	TTGGGCAGGTGCTGTTGAT	TGCCCACTTCTGCAACGA
GUSB	CGCCCTGCCTATCTGTATTC	TCCCCACAGGGAGTGTGTAG
ChIP-qPCR		
**Name**	**Forward 5´**	**Reverse 3´**
FR	CAGCGACCTCGTGAATATGA	AAACCACTTGCCCACAAAAC
DS	ATGTAAATAAAACCGTGACAGCTCAT	TTACCCAACGGGAAGCATATG
Qp	AAATTGGGTGACCACTGAGGGAGT	ATAGCATGTATTACCCGCCATCCG
OriLyt	TCGCCTTCTTTTATCCTCTTTTTG	CCCAACGGGCTAAAATGACA
LF3	TCCGGTCACGTCTCATGTTG	AAGGCAGCCAGGTGGATTTT
GADPH	CGGTGCGTGCCCAGTT	CTACTTTCTCCCCGCTTTTTTTT
IL6R	AGGTGTTTAGGTGCCATCCACAGA	GAGGCAAGGAAACGGTTTGGACTT
GKN1/2	TGTTGCACTCCTTTCCCAAAGCTG	ACTTCAAGGGTAGCCCAAAGACCT
CDC6	CCTGGAGGAAGAAGGGACAGATTT	ACCTTGTAGTCTAACTGCAAGGCGCA
FAM55B	ACACACAGCACAGCCACCAT	GCTGATCCCCCCTGCAGTA
SELK	CGACTTGATCGTGCAGAGTTGGTT	TGTAGTCAGCACCCTCAAACGTCA

siRNA		
**Name**	**Sequence**	
siControl	UGGUUUACAUGUCGACUAA<br>UGGUUUACAUGUUGUGUGA<br>UGGUUUACAUGUUUUCUGA	

	UGGUUUACAUGUUUUCCUA	
SiH2A.Z	UCUAAAGGAUGCCUGGAUU<br>GAUGCAGAAGUUAUAGUAA<br>CAACCAAAUUUCUGCAUUC<br>CAAUAAAGGUCAUAUCCCA	
shRNA		
**Name**	**Sense sequence**	**Full Hairpin Sequence**
shH2A.Z-A	GCTTCAAAGAAGCTATTGATT	CCGGGCTTCAAAGAAGCTATTGATTCTCG<br>AGAATCAATAGCTTCTTTGAAGCTTTTTG
shH2A.Z-B	CGTGGAGATGAAGAATTGGAT	CCGGCGTGGAGATGAAGAATTGGATCTCG<br>AGATCCAATTCTTCATCTCCACGTTTTTG
